# Complete closure of a large gastric defect after endoscopic submucosal dissection by double-layered suturing using wide-opening reopenable clips

**DOI:** 10.1055/a-2749-3411

**Published:** 2025-12-11

**Authors:** Yusuke Takahashi, Kotaro Shibagaki, Mayu Kawamoto, Shinsuke Suemitsu, Satoshi Kotani, Norihisa Ishimura, Shunji Ishihara

**Affiliations:** 1 175764 Department of Gastroenterology, Faculty of Medicine, Shimane University, Izumo, Japan

A man in his 70s underwent en bloc endoscopic submucosal dissection (ESD) for two early gastric cancers on the lesser curvature of the gastric body, yielding a single 55 × 30 mm specimen. Prophylactic closure was performed using a double-layered suturing technique 1 2 with wide-opening SureClips (16 mm; Micro-Tech, Nanjing, China) to prevent delayed bleeding ( Fig. 1 ).

**Fig. 1:**
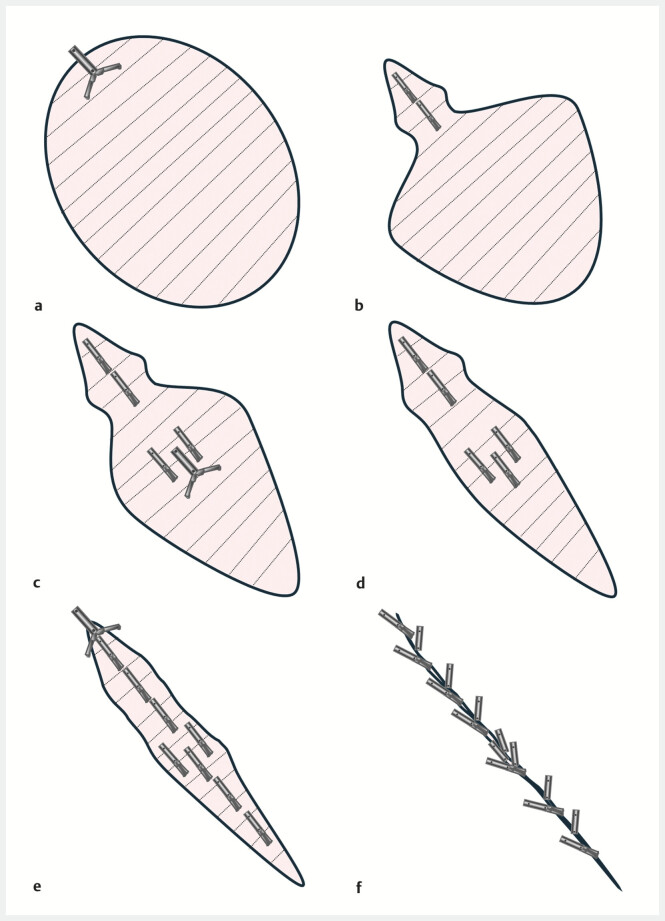
Schematic of double-layered suturing for closure of a mucosal defect after gastric ESD. **a** The muscularis propria was grasped with SureClips and inverted toward the lumen. **b** The muscular layer was then approximated sequentially from distal to proximal, which progressively reduced the defect. **c** In large defects, two parallel lines of muscular infolding were created by clipping, which markedly shortened the defect in the short-axis direction. **d** Muscular flaps were infolded by additional clips, further reducing the defect and eliminating dead space. **e** After completing muscular infoldings, to the oral side, a slit-like, long-axis mucosal gap remained, and closure was sequentially performed from the distal to the oral side. **f** Complete closure was achieved. ESD, endoscopic submucosal dissection.

All steps were performed in a forward view under minimal insufflation to maintain gastric wall relaxation. The muscularis propria was grasped with clips and inverted toward the lumen, followed by sequential distal-to-proximal approximation to gradually reduce the defect. In the widest part of the mucosal defect, two parallel lines of muscular infolding were created to further shorten the gap. After muscular infoldings, the mucosal edges were approximated mucosa-to-mucosa. Complete closure was achieved with 16 clips in 22 minutes. No delayed bleeding was observed, and follow-up endoscopy at 4 weeks confirmed complete re-epithelialization ( Fig. 2 , Video 1 ).

**Fig. 2:**
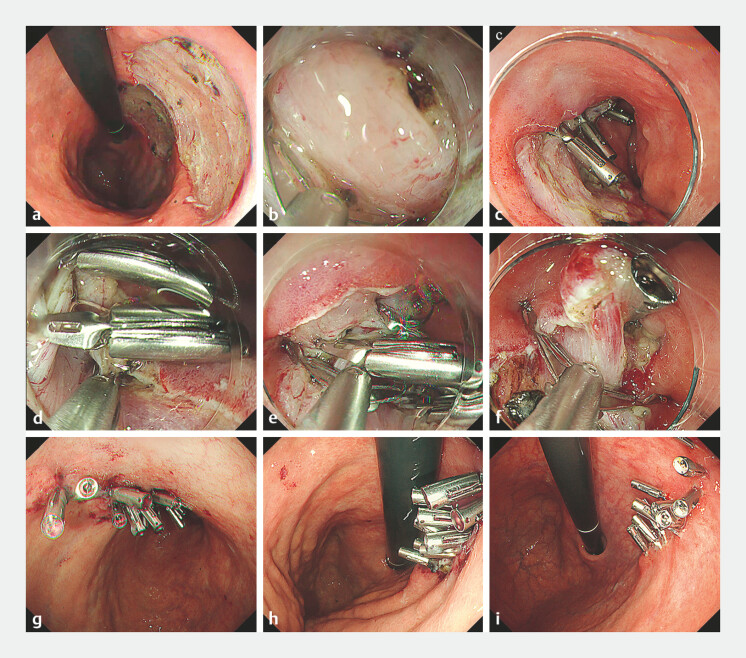
Endoscopic course in the present case. **a** The two ESD resection fields merged, resulting in a single large mucosal defect. To prevent delayed bleeding, prophylactic closure by double-layered suturing was performed. **b** In a forward view with minimal insufflation, the muscularis propria was grasped and infolded by SureClip from the distal side. **c** Muscular infolding reduced the mucosal defect. **d** In large defects, two parallel lines of muscular infolding were created by clipping. **e** Two muscular flaps were folded with an additional clip, resulting in further shortening of the defect along the short-axis. **f** After completing muscular infoldings, the approximated mucosal edges were then clipped in a mucosa-to-mucosa fashion. **g, h** Complete closure was achieved with 16 clips in 22 minutes. **i** The closure remained secure and complete re-epithelialization was confirmed 4 weeks later. ESD, endoscopic submucosal dissection.

Double-layered suturing of a post-gastric ESD defect using reopenable clips and its clinical course. ESD, endoscopic submucosal dissection.Video 1

Prophylactic closure after gastric ESD reduces the risk of delayed bleeding 3 . Simple mucosa-to-mucosa closure is challenging for large defects and often leaves a submucosal cavity. To overcome this limitation, several methods, including the reopenable clip-over-the-line method 3 , endoscopic ligation with O-ring closure 4 , and endoscopic hand suturing 5 , have been developed, but they remain complex and time-consuming. Double-layered suturing provides a simple, rapid, and cost-effective alternative with minimal dead space and proven utility after colorectal and duodenal ESD 1 2 . However, its use for large gastric defects, where clipping is difficult because of the thick gastric wall, has rarely been described. This case demonstrates the successful closure of a large post-ESD defect on the lesser curvature of the gastric body – a site difficult to approximate – using wide-opening SureClips under minimal insufflation in a forward view. This technique may help prevent delayed bleeding and promote faster epithelial healing after large gastric ESD.

Endoscopy_UCTN_Code_TTT_1AO_2AG

## Notes

Endoscopy E-Videos https://eref.thieme.de/e-videos
*E-Videos* is an open access online section of the journal *Endoscopy* , reporting on interesting cases and new techniques in gastroenterological endoscopy. All papers include a high-quality video and are published with a Creative Commons CC-BY license. Endoscopy E-Videos qualify for HINARI discounts and waivers and eligibility is automatically checked during the submission process. We grant 100% waivers to articles whose corresponding authors are based in Group A countries and 50% waivers to those who are based in Group B countries as classified by Research4Life (see: https://www.research4life.org/access/eligibility/ ). This section has its own submission website at https://mc.manuscriptcentral.com/e-videos .
